# Phase Engineering of Nanomaterials: Tailoring Crystal Phases for High-Performance Batteries and Supercapacitors

**DOI:** 10.3390/mi16111289

**Published:** 2025-11-16

**Authors:** Ramanadha Mangiri, Nandarapu Purushotham Reddy, Joonho Bae

**Affiliations:** 1 Department of Physics and Semiconductor Science, Gachon University, Seongnam-si 13120, Gyeonggi-do, Republic of Korea; ramanadh@gachon.ac.kr; 2 Department of Electronic Engineering, Yeungnam University, 280 Daehak-Ro, Gyeongsan 38541, Gyeongbuk, Republic of Korea; npurush1992@gmail.com

**Keywords:** phase engineering, transition metal dichalcogenide, Li-ion batteries, Li-S batteries, supercapacitors

## Abstract

Phase engineering has emerged as a powerful method for manipulating the structural and electrical characteristics of nanomaterials, resulting in significant enhancements in their electrochemical performance. This paper examines the correlation among morphology, crystal phase, and electrochemical performance of nanomaterials engineered for high-performance batteries and supercapacitors. The discourse starts with phase engineering methodologies in metal-based nanomaterials, including the direct synthesis of atypical phases and phase transformation mechanisms that provide metastable or mixed-phase structures. Special emphasis is placed on the impact of these synthetic processes on morphology and surface properties, which subsequently regulate charge transport and ion diffusion during electrochemical reactions. Additionally, the investigation of phase engineering in transition metal dichalcogenide (TMD) nanomaterials highlights how regulated phase transitions and heterophase structures improve active sites and conductivity. The optimized phase-engineered ZnCo_2_O_4_@Ti_3_C_2_ composite exhibited a high specific capacitance of 1013.5 F g^−1^, a reversible capacity of 732.5 mAh g^−1^, and excellent cycling stability, with over 85% retention after 10,000 cycles. These results confirm that phase and morphological control can substantially enhance charge transport and electrochemical durability, offering promising strategies for next-generation batteries and supercapacitors. The paper concludes by summarizing current advancements in phase-engineered nanomaterials for lithium-ion, sodium-ion, and lithium-sulfur batteries, along with supercapacitors, emphasizing the significant relationship between phase state, morphology, and energy storage efficacy. This study offers a comprehensive understanding of the optimal integration of phase and morphological control in designing enhanced electrode materials for next-generation electrochemical energy storage systems.

## 1. Introduction

**Figure 1:**
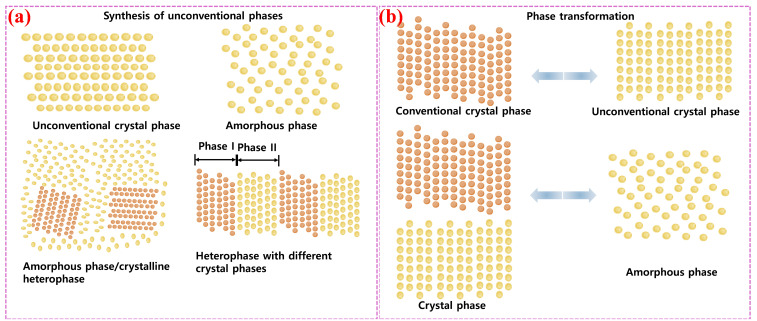
PEN consists of (**a**) the direct synthesis of unconventional phases and (**b**) the phase transformation of nanomaterials.

Phase engineering of nanomaterials (PENs) has emerged as a practical approach to developing new electrode materials for electrochemical energy storage devices, including supercapacitors and batteries. To date, research on phase-engineered nanomaterials (PENs) has led to the development of several new and unusual phases, including unconventional crystal structures, amorphous phases, and mixed phases that contain two or more coexisting phases (Figure 1a). As shown in Figure 1b, phase transformation offers a valuable method for creating unconventional phases from normal ones by applying specific conditions, such as high temperatures or high pressures [1,2]. PEN focuses on modifying atomic configurations, polymorphs, and crystallinity of materials at the nanoscale. These aspects directly influence electronic structure, ion transport pathways, and electrochemical behavior. Historically, nanomaterials were mainly produced in their thermodynamically stable phases. However, advances in nanotechnology and synthetic techniques now enable the stabilization of unconventional or metastable phases that are rarely observed in bulk materials [1,2]. Metastable or mixed phases often demonstrate enhanced physicochemical properties, such as higher conductivity, catalytic activity, and ion transport, due to their distinct atomic coordination and defect structures.

In nanoscale materials, surface and interface energies dominate bulk free energy. This dominance helps stabilize non-equilibrium phases that cannot be seen in bulk forms [3]. Because of surface energy, it is possible to create materials with unique crystal structures. Examples include 1T and 2H phases in MoS_2_, 4H phases in noble metals, and amorphous or crystalline heterophases in oxides [4,5]. Transition metal dichalcogenides (TMDs), such as MoS_2_ and WS_2_, can transition from a semiconducting 2H phase to a metallic 1T or mixed 1T/2H phase. This switch is done with chemical exfoliation or intercalation techniques. The metallic 1T phase offers improved electronic conductivity and a higher density of active sites. As a result, it enhances rate capability and charge storage in supercapacitors or lithium/sodium-ion batteries [6,7]. Similarly, metal oxides and hydroxides, such as NiCo_2_O_4_, Fe_2_O_3_, and MnO_2_, show a strong dependence of electrochemical performance on their phase [8,9]. For example, amorphous or poorly crystalline phases have more accessible redox-active sites and flexible atomic networks for ion transport. In contrast, crystalline phases provide stability during repeated cycling [10,11].

Combining these forms to create amorphous/crystalline heterophase structures enhances cycling stability and capacitance. This benefit results from the synergistic effects of conductivity and ion accessibility [12]. Heterophase junctions in nanomaterials can generate intrinsic electric fields that improve charge transfer and reduce recombination. Consequently, electrochemical performance is improved [13]. Phase engineering also applies to metal–organic frameworks (MOFs) and their derivatives. Processes like partial amorphization or polymorphic transitions during pyrolysis produce hierarchical porous structures and improved ion diffusion [14]. Adjusting the phase composition in these materials helps optimize the balance between electron and ion transport, which is vital for supercapacitor and battery electrodes. Despite these advances, challenges persist in large-scale synthesis and maintaining the stability of metastable phases during extended cycling. Ongoing efforts in operando characterization and theoretical modeling are crucial for understanding phase evolution during electrochemical processes. These efforts guide the rational design of stable, high-performance electrodes [15,16].

PEN has become an essential method for customizing physicochemical properties and enhancing material performance in energy storage applications. Managing crystal phases, phase boundaries, and structural transformations allows PEN to adjust electronic structures, defect chemistry, ion diffusion paths, and interfacial stability. Crystal phase regulation plays a pivotal role in optimizing the electronic structure, defect density, and ion diffusion pathways of electrode materials. By controlling the crystal phase, the coordination environment, and oxidation states of the metal centers can be precisely tuned, thereby influencing both electrical conductivity and redox activity. For example, metastable or mixed phases often exhibit enhanced charge carrier mobility and higher surface energy, which facilitate faster ion/electron transport and provide more active sites for redox reactions. Additionally, phase boundaries formed during phase engineering serve as internal heterointerfaces, promoting charge separation and minimizing recombination losses. Consequently, materials with engineered crystal phases show significantly improved specific capacitance, rate capability, and cycling stability compared to their single-phase counterparts. This control is beneficial for supercapacitors and lithium-ion batteries [17,18,19,20]. The phase composition has a significant influence on energy density, power output, and cycling stability. Recent advances in creating unconventional phases in metals, metal oxides, chalcogenides, and various hybrid systems have led to the development of new designs for high-performance electrode materials with enhanced charge storage and transport capabilities [20,21,22,23]. This review covers the latest progress in phase-engineered nanomaterials for electrochemical energy storage, emphasizing core mechanisms, material design strategies, and the main challenges facing next-generation supercapacitors and Li-ion batteries.

## 2. Phase Engineering in Metal Nanomaterials

### Direct Synthesis of Unconventional Phase Metal Nanomaterials

In recent decades, researchers have extensively studied how to control the structure of metal nanomaterials to modify their properties and performance. Recently, a significant focus has been placed on phase engineering of metal nanomaterials, leading to the discovery and controlled synthesis of many new materials with unconventional crystal phases. To date, these unusual metal nanostructures can be directly prepared from metal precursors using various methods such as wet-chemical reduction, seed-mediated growth, template-assisted synthesis, and thermal annealing. In this section, we briefly describe these synthesis techniques and highlight some representative examples of the direct fabrication of unconventional-phase metal nanomaterials (Table 1).

The wet-chemical (colloidal) reduction method is versatile and widely used for producing metal nanomaterials. It operates under mild conditions with simple equipment. Metal salts are reduced in solution using chemicals like NaBH_4_, hydrazine, or ascorbate. Surfactants, ligands, or capping agents prevent particles from clustering and can also shape the materials into various forms, such as cubes, rods, or plates. Adjusting factors such as temperature, the type and number of precursors, the strength of the reductant, the kind and quantity of surfactant, the solvent, and the sequence and timing of mixing influences both the energy driving the reaction and the barriers to forming new structures. Consequently, solution reduction enables researchers to create or maintain unusual material phases.

For instance, Fan et al. employed sequential ligand control and growth kinetics to stabilize a 4H hexagonal Au nanoribbon structure in solution. This nanoribbon served as a seed for the subsequent phase control of overgrown metals [26]. In a similar vein, metastable intermetallics like Au_3_Fe, Au_3_Co, or Au_3_Ni have been synthesized through low-temperature solution methods. Researchers achieved these forms by meticulously managing reduction potential and ligand environment [37]. Nonetheless, the difficulty in wet-chemical reduction lies in the tendency of metastable phases to transition to their thermodynamically stable bulk phase. This transformation is especially challenging when subjected to elevated temperatures or extended reaction durations. Approaches like rapid quenching, the application of potent binding ligands, or kinetic trapping through capping layers are frequently essential for preserving the metastable structure.

**Figure 2:**
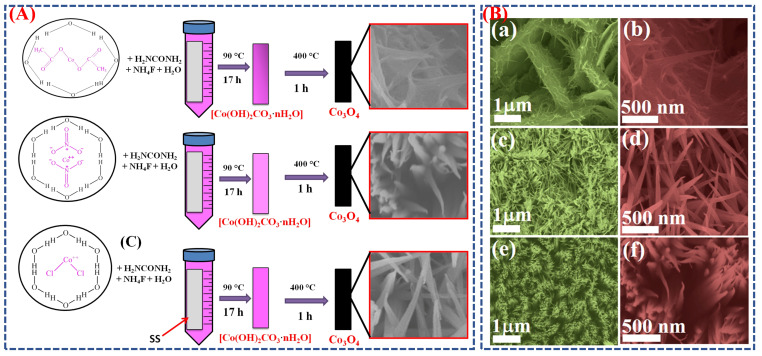
(**A**) Schematic representation of the preparation of CA-CO_3_O_4_, CN-Co_3_O_4,_ and CC-Co_3_O_4_ electrodes. (**B**)—(**a**–**f**) Low and high-magnification FE-SEM images of CC-CO_3_O_4_ and CN-Co_3_O_4_ (reproduced with permission [38]).

Gaikar et al. developed Co_3_O_4_ electrodes with various structures using a cheap, simple wet-chemical process called chemical bath deposition [38]. Figure 2A illustrates the stepwise chemical bath deposition process used for the synthesis of Co_3_O_4_ nanostructures from different cobalt precursors. Initially, cobalt salts undergo hydrolysis and nucleation to form cobalt hydroxide intermediates, which subsequently transform into Co_3_O_4_ through controlled annealing. Each precursor type affects ion release kinetics and crystal growth, resulting in distinct morphologies such as nano-platelets (acetate), nano-needles (chloride), and nano-grass-like structures (nitrate). The schematic highlights these transformation pathways, connecting precursor chemistry to the final morphology and electrochemical performance. FESEM images (Figure 2B(a–f))confirmed these morphological differences. Cobalt acetate yielded nano-platelets with fine nano-needles; cobalt chloride produced randomly spread nano-needles; and cobalt nitrate created a uniform, highly porous nano-grass-like structure. This porous formation offers a large surface area, facilitating ion diffusion and thereby enhancing electrochemical performance. The nano-grass Co_3_O_4_ electrode achieved a specific capacity of 66.4 mAh g^−1^ at a current density of 1 mA cm^−2^, exhibiting excellent cyclic stability (~90% retention after 1000 cycles) and low internal resistance. These results show that wet-chemical synthesis enables precise control over morphology, which directly enhances electrochemical behavior [39]. Overall, the schematic and morphological images demonstrate how this method effectively adjusts structure and surface area, leading to improved supercapacitor performance of Co_3_O_4_ electrodes.

Seed-mediated or epitaxial growth is an effective technique for developing shells that either conform to or are influenced by the phase of a core seed. Imagine that the shell metal can grow coherently (or semi-coherently) on the core with only a manageable lattice mismatch. In this case, the shell may adopt the nonstandard crystal stacking or the metastable polymorph of the core. Fan et al. elegantly demonstrated this by using 4H hexagonal Au nanoribbons as seeds and growing shells of Ir, Rh, Os, Ru, and Cu through solution-phase epitaxial growth. This produced 4H/fcc polytypic Au@M core–shell nanoribbons. The Au core partially transformed from 4H to fcc during coating, but the epitaxial relationship stayed intact. This led to alternating 4H and fcc domains within the shell. Recent advances have employed a similar strategy on Pd-based alloys, such as PdFe, PdIr, and PdRu, utilizing 4H Au seeds [40]. This produced unconventional 4H-phase cores/shells with improved electrocatalytic properties. Seed-mediated epitaxy offers structural coherence, phase templating, and control over morphology. However, limitations include lattice mismatch, interface strain, and the risk of phase relaxation if the shell grows too thick.

**Figure 3:**
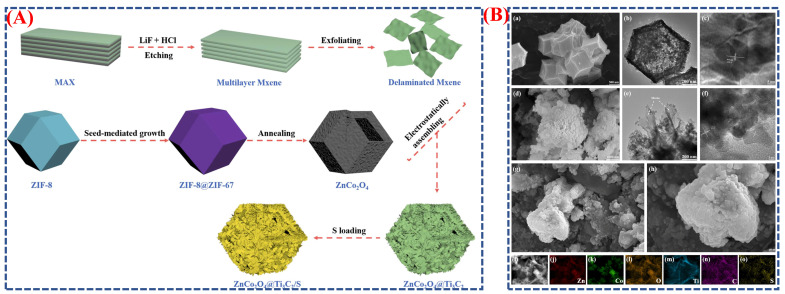
(**A**) Schematic process of the ZnCo_2_O_4_@Ti_3_C_2_/S material (**B**) (**a**–**o**) SEM, TEM, and HRTEM and STEM EDS elemental mapping images of ZnCo_2_O_4_@Ti_3_C_2_/S material (reproduced with permission [38]).

Wei et al. developed a ZnCo_2_O_4_@Ti_3_C_2_ composite derived from MOF using a seed-mediated growth technique and controlled heat treatment [38]. The process begins with the creation of ZIF-8@ZIF-67 core–shell structures, where ZIF-8 serves as the core and ZIF-67 as the shell (Figure 3A). Calcination then transforms this into hollow ZnCo_2_O_4_ polyhedra. Next, MXene (Ti_3_C_2_) nanosheets are electrostatically deposited onto the ZnCo_2_O_4_ surface, forming the ZnCo_2_O_4_@Ti_3_C_2_ hybrid (Figure 3B(a–o)). Morphological images reveal that ZnCo_2_O_4_ maintains a consistent polyhedral shape with a hollow interior and a textured surface, which facilitates sulfur loading and ion transport. The MXene coating improves electrical conductivity and stability without altering the overall shape. This engineered composite demonstrates a high initial discharge capacity of 1283.9 mAh g^−1^ and excellent cycling stability over 400 cycles. The schematic and morphology analyses highlight how the seed-mediated method provides precise structural control, enhancing electrochemical performance. This strategy shows promise for advanced batteries and supercapacitors.

Templating generally helps stabilize or direct the growth of unconventional phases by applying constraints or interfaces. It can maintain nonstandard phases through mechanisms like interfacial strain, confinement, or chemical interactions, even without crystal lattice continuity. For example, two-dimensional supports such as graphene oxide (GO), functionalized carbon, metal oxides, or other nanoscale hosts have promoted the formation of unique metal phases on their surfaces. A template can prevent aggregation, provide nucleation sites, or restrict atomic rearrangement. Several specific examples of unconventional-phase metals grown on GO are available in the references reviewed so far, although templating approaches are also discussed in the literature.

**Figure 4:**
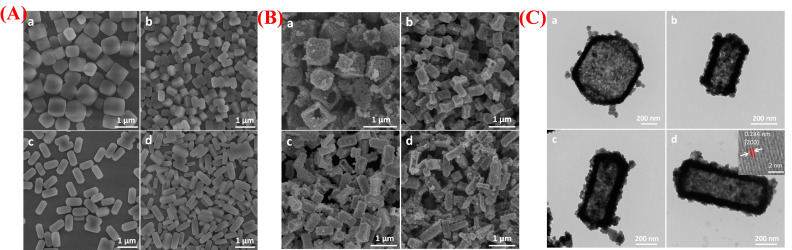
FE-SEM images of Ni complex (**A**) (**a**–**d**), NiS_2_ hollow prisms (**B**) (**a**–**d**), and (**C**) (**a**–**d**) TEM images of NiS_2_ hollow prisms (reproduced with permission [41]).

Dai et al. developed NiS_2_ hollow prisms using a template-assisted synthesis method that employed a nickel complex as the sacrificial template [41]. Figure 4A(a–d) illustrates the process of creating these NiS_2_ hollow prisms, where nickel acetate hydroxide complexes serve as templates. During the reflux reaction with thioacetamide, the nickel complexes gradually convert into NiS_2_ hollow prisms via the Kirkendall diffusion mechanism. This templating approach allows for precise control over the shape and size of the final NiS_2_ structures. FESEM images (Figure 4B(a–d)) and TEM images (Figure 4C(a–d)) confirm that the synthesized NiS_2_ materials consistently form hollow prisms, maintaining the structure of the original nickel complex templates. TEM analysis shows that the hollow shells consist of densely packed NiS_2_ nanoparticles, providing a large surface area and enabling fast ion and electron transport. Thanks to this unique hollow structure, the NiS_2_ electrode exhibits excellent electrochemical performance, achieving a specific capacitance of 1725 F g^−1^ at 5 A g^−1^ and maintaining 1193 F g^−1^ at 40 A g^−1^. The material also demonstrates outstanding cycling stability, with capacitance increasing from 1367 F g^−1^ to 1680 F g^−1^ after 10,000 cycles. The schematic and morphological analyses clearly show that the templated synthesis method is a practical approach for producing hollow nanostructures with controlled shape and enhanced electrochemical properties. Overall, the NiS_2_ hollow prisms are a promising electrode material for high-performance supercapacitors.

Electrochemical techniques, such as pulsed electrodeposition and partial electrochemical reduction, offer advantageous pathways for creating metastable metal phases. These methods operate under ambient conditions, allowing precise control over the timing of nucleation and growth. An example is the pulsed electrodeposition of metastable intermetallic Pd31Bi12 nanoparticles supported on carbon [42]. High-overpotential pulses promote uniform nucleation, while lower potentials facilitate growth. The authors achieved ordered intermetallic structures at the nanoscale under mild aqueous conditions. This method avoids the need for high-temperature solid-state synthesis. In electrochemical synthesis, pulse parameters (amplitude, width, and duty cycle), ion concentrations, substrate type, and electrolyte pH are often noted to influence composition, phase, and crystalline order.

Another example is the partial electrochemical reduction of crystalline SnS_2_ nanosheets, as demonstrated by cyclic voltammetry. This reduction forms amorphous Sn regions within the crystalline SnS_2_ matrix. The process results in heterophase Sn/amorphous Sn–SnS_2_ nanostructures. The amount of the amorphous phase can be adjusted by changing the electrolyte pH and cycling protocol. In summary, electrodeposition and reduction provide significant advantages. They are compatible with ambient conditions, easy to operate, and enable precise timing. Challenges remain in controlling uniformity, preventing overgrowth, balancing reduction and dissolution, and ensuring phase purity.

**Figure 5:**
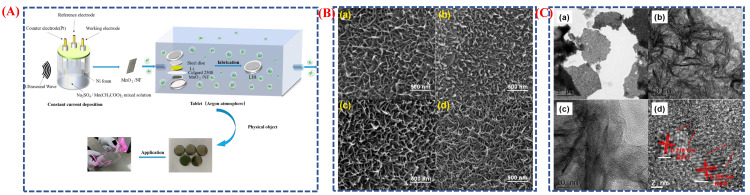
(**A**) Schematic representation of electrode preparation and cell fabrication, (**B**) (**a**–**d**) SEM images of MnO_2_, (**C**) (**a**–**d**) TEM, HRTEM images of MO_7_ (reproduced with permission [43]).

Dai et al. developed MnO_2_ electrodes using ultrasonic-assisted electrodeposition (templated synthesis) to investigate the influence of different current densities on their structure and electrochemical properties [43]. Figure 5A presents a schematic of the electrode fabrication and cell assembly process, where MnO_2_ was deposited on nickel foam at various current densities. The nickel foam served as a three-dimensional conductive scaffold, supporting uniform growth of MnO_2_ and facilitating ion and electron transport. TEM and HR-TEM images Figure 5C(a–d) showed interconnected nanorods of 10 to 20 nm, with lattice fringes matching the (002) and (211) planes of MnO_2_, indicating high crystallinity. These nanorods created a porous network that improved ion mobility and charge transfer during electrochemical reactions. SEM images (Figure 5B(a–d)) show that the surface morphology varies with current density: low currents yield broad, irregular grains, while higher currents produce fine nanorod networks with tip aggregation, thereby increasing the surface area and the number of active sites. The study demonstrated that refined morphology enhanced charge storage and conductivity. The best-performing sample, prepared at seven mA cm^−2^, showed a specific capacitance of 415.4 F g^−1^ at 1 A g^−1^ and achieved a lithium-ion battery capacity of 818.1 mAh g^−1^ after 200 cycles. The schematics and images illustrate that templated electrodeposition enables precise control over structure and morphology, boosting performance in supercapacitors and batteries. This research underscores that combining template-assisted design with controlled synthesis can produce highly active, nanostructured electrodes.

## 3. Phase Transformation in Metal Nanomaterials

### 3.1. Thermal Activation

In metal nanomaterials, thermal activation is a fundamental thermodynamic method for inducing atomic rearrangements and structural development. Phases may go from metastable to stable with controlled heating. The 4H-to-fcc phase transition of Au nanoribbons during annealing, for example, has been observed using in situ transmission electron microscopy (TEM). Despite a slight “Rayleigh instability” below 400 K, the 4H phase was mainly preserved, demonstrating its thermal durability when exposed to electron beams [26]. An analogous method for creating amorphous-to-crystalline transitions in noble metal nanosheets is annealing-induced phase development [44].

**Figure 6:**
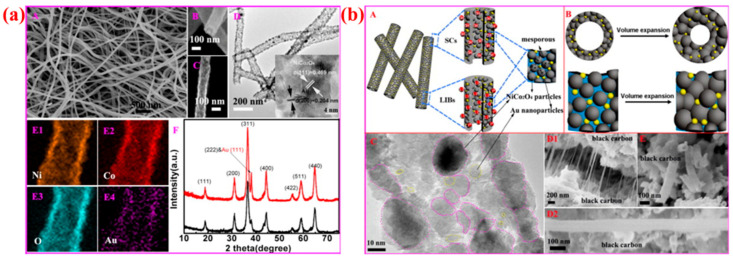
(**a**) (**A**–**F**) SEM, XRD patterns, (**b**) (**A**–**E**) TEM images of NiCo_2_O_4_@Au at different magnifications (reproduced with permission [45]).

Jian Zhu et al. demonstrated the phase transformation and structural evolution in NiCo_2_O_4_ nanotubes decorated with ultrafine Au nanoparticles (NiCo_2_O_4_@Au NTs) [45], as illustrated in Figure 6a,b. They prepared these nanostructures using a simple electrospinning method, followed by calcination, which resulted in uniform, hollow, and mesoporous nanotubes. The schematic diagram (Figure 6b) shows the well-organized one-dimensional nanotube structure with evenly distributed Au nanoparticles. The addition of ultrafine Au nanoparticles played a key role in modifying the phase structure and enhancing the overall electrochemical performance. During the transformation from precursor nanofibers to crystalline NiCo_2_O_4_@Au nanotubes, the morphology was well retained while the Au nanoparticles acted as conductive and mechanical connectors between NiCo_2_O_4_ grains. This phase-transformation-driven morphology stabilization improved charge transfer and mechanical stability. Figure 6a schematically explains how Au nanoparticles strengthen the NiCo_2_O_4_ framework during charge/discharge cycles and alleviate volume changes through a flexible, interconnected network. These features resulted in excellent electrical conductivity, high pseudocapacitance, and superior cyclic stability in both supercapacitor and lithium-ion battery applications. The authors achieved a high specific capacitance of 1013.5 F g^−1^ with 85.13% retention after 10,000 cycles and a reversible capacity of 732.5 mAh g^−1^ after 200 cycles. Overall, this study emphasizes the importance of phase transformation control and structural engineering in metal nanomaterials. Uniform Au nanoparticle decoration not only induces conductive phase evolution but also stabilizes morphology, leading to exceptional electrochemical behavior, as clearly shown in Figure 6a,b of their schematic explanation.

### 3.2. High Pressure

Pressure-driven phase transitions can be triggered by effectively decreasing interatomic distances through the application of high pressure. Researchers have observed the structural changes of metal nanoparticles during compression using in situ synchrotron X-ray diffraction with a diamond-anvil cell. For example, pressure-induced transformations have been shown in Au, Fe, and Pt systems, leading to the discovery of previously unknown metastable structures [46,47]. These studies show how lattice symmetry and electronic configuration are adjusted by strain and pressure.

**Figure 7:**
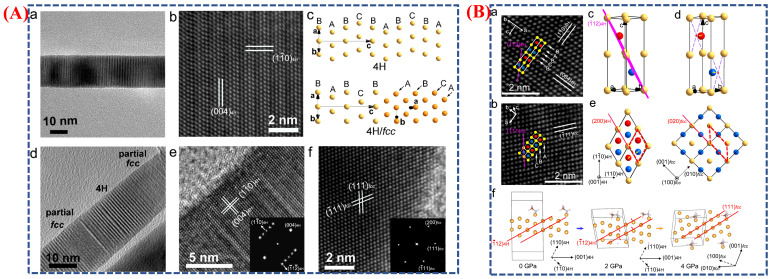
(**A**) (**a**–**f**) TEM and HRTEM images of as-synthesized 4H Au NRBs before compression and after recovery from 14.3 GPa to 1 atm. The schematic illustration displays the atomic arrangements of single-phase 4H and heterostructured 4H/fcc Au NRBs. TEM and HRTEM images of recovered samples reveal the coexistence of 4H and fcc phases. (**B**) (**a**–**f**) Proposed 4H-to-fcc phase transition mechanism of Au NRBs based on HRTEM and calculations (reproduced with permission [48]).

The HRTEM image of the as-synthesized 4H Au NRB shows differently colored Au atoms marking the stacking layers (A—red, B—yellow, C—blue). After pressure treatment, the recovered Au NRB exhibits an fcc structure. Schematics illustrate the 4H unit cell, the ( $\bar{11}$2)4H plane, and the transition process as viewed along the (001) 4H and (100) fcc planes [48], with red dashed lines indicating the same atomic positions before and after compression. Calculated structures at 0, 2, and 4 GPa demonstrate the pressure-induced structural evolution of 4H Au with methylamine-covered (110) surfaces. Gold (Au) nanoribbons (NRBs) undergo a pressure-induced transition from a hexagonal 4H phase to a face-centered cubic (fcc) phase, demonstrating the necessity of research on phase changes in metal nanomaterials. The atomic arrangements in the 4H and fcc structures, along with the coexistence of these phases after high-pressure treatment, are clearly illustrated in the schematic and microscopic images shown in Figure 7A(a–f). The 4H Au nanoribbons were created by scientists using a wet-chemical method and then exposed to increasing external pressures up to 14.3 GPa. Atomic rearrangements and layer sliding within the gold lattice led to a gradual shift in the crystal structure from 4H to fcc under compression. The irreversible nature of this phase transition indicates that the fcc phase is more thermodynamically stable under these conditions. The schematic illustration in Figure 7B(a–f) further explains the atomic-scale mechanism underlying this change. It shows that Au atoms are in the 4H phase.

### 3.3. Surface Modification

The high surface-to-volume ratio at the nanoscale makes surface energy the primary factor. It is therefore possible to stabilize or destabilize certain crystal phases by changing surface ligands or adsorbates. The ligand exchange from oleylamine to octadecanethiol in Au nanosheets causes the 2H-to-fcc transition, which is a prominent example of a phase transition. Rearrangement toward the fcc structure is promoted by this process, which alters surface binding energies [49]. In PdCu and Pt nanostructures, surface-controlled phase switching has also been demonstrated through etching and surface adsorption [50].

**Figure 8:**
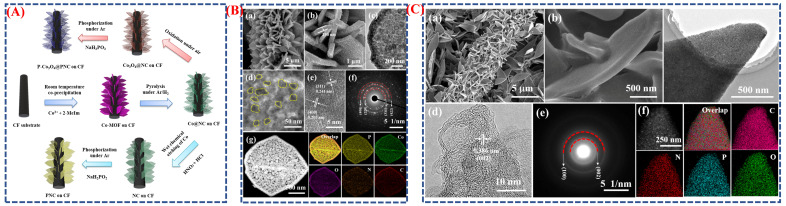
(**A**) Schematic figure of the synthesis of the P-Co3O4@PNC and PNC nanosheets on the CF substrate; (**B**) (**a**–**g**) SEM, TEM images, and STEM EDS mapping images of P–Co_3_O_4_@PNC; (**C**) (**a**–**f**) SEM, TEM, and HRTEM, and EDS mapping images of PNC nanosheets (reproduced with permission [51]).

Liu et al. conduct a comprehensive analysis of phase-engineered MOF-derived cobalt-based hybrid nanosheets and their influence on the morphology and electrochemical performance of supercapacitors [51]. Phase engineering is essential for optimizing the surface, electrical, and structural characteristics of electrode materials. It enables precise modification of crystal phases and defect concentrations, hence altering redox activity, charge transfer, and ion diffusion. Altering the phase and composition of materials can enhance conductivity, increase the number of active sites, and improve stability during the cycling process. Figure 8A illustrates the authors’ schematic synthesis pathway for P–Co_3_O_4_@PNC and PNC nanosheets grown on carbon fiber (CF). Initially, Co-MOF is synthesized directly on CF. Subsequently, it undergoes carbonization, oxidation, and phosphatization. This procedure produces P-N co-doped carbon nanosheets that encase phosphorus-doped CoO_4_ nanoparticles. This significantly enhances the mechanical flexibility and charge conduction of the material. The progression from Co–MOF to Co_3_O_4_ and ultimately to P–Co_3_O_4_@PNC illustrates the significance of structural engineering in maintaining the integrity of nanosheets and creating surfaces with many flaws that facilitate ion mobility.

Figure 8B(a–g) illustrates SEM, TEM, and HRTEM images that verify the consistent nanosheet shape, which is distinguished by a high density of perforations and ultrafine P–Co_3_O_4_ nanoparticles (~20 nm) that are incorporated into the carbon matrix. The electrochemical activity is enhanced by the porous and linked architecture, which provides a vast surface area and minimizes ion diffusion distances. The HRTEM study suggests that P doping induces lattice distortions, which in turn increase electron mobility and defect density. The morphological advantages that result from phase engineering are directly linked to enhanced electrochemical performance, as they facilitate rapid charge–discharge reactions and improve electrode stability during cycling. Figure 8C(a–f) provides a detailed explanation of the manufacturing and electrochemical evaluation of flexible solid-state asymmetric supercapacitor (ASC) devices. The positive electrode is P–Co_3_O_4_@PNC, while the negative electrode is PNC. The hybrid structure’s exceptional rate performance, extraordinary cycling stability, and high capacitance (198 F g^−1^ at 1 A g^−1^) are all validated by the schematic design and electrochemical findings, which demonstrate 96.8% capacitance retention after 10,000 cycles. The ASC device demonstrates exceptional mechanical durability and flexibility, ensuring it can power an LED light while maintaining performance in various bending scenarios.

### 3.4. Secondary Growth

Phase changes can also occur through secondary growth or epitaxial deposition on pre-existing nanostructures due to strain and interfacial mismatch. For example, 2H/fcc heterophase Au nanoplates were formed by depositing Au on 2H-Au nanosheets, where the fcc regions nucleated at critical thicknesses driven by surface energy minimization [52]. The lattice mismatch between core and shell metals may also lead to 2H-to-fcc conversion when Ag, Pt, or Pd epitaxially overgrows on Au nanosheets [53].

**Figure 9:**
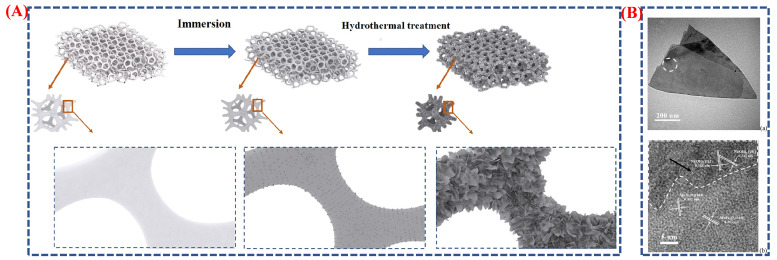
(**A**) Flow chart of the synthesis preparation of MFO-NSAs (**B**) TEM, HRTEM images of MFO-NSAs (reproduced with permission [54]).

The epitaxial development of MnFeO_4_ nanosheet arrays (MFO–NSAs) on nickel foam was effectively demonstrated by the authors of this paper, Mingjie Fei et al., using a hydrothermal and immersion approach, as illustrated in Figure 9A,B [54]. The epitaxial growth of MnFeO_4_ nanosheet arrays (MFO–NSAs) on nickel foam serves as an example of phase engineering, where the lattice-matched interface between MnFeO_4_ and the Ni substrate promotes preferential crystal orientation and stabilizes a specific phase configuration. This controlled epitaxial interaction tailors the local electronic structure, enhances charge transport efficiency, and reduces interfacial resistance—hallmark features of phase-engineered nanomaterials (PENs). The pretreatment Ni foam was submerged in a pH 13 precursor solution throughout the synthesis process, which caused Ni(OH)_2_ seed layers to develop in situ. Under the guidance of a minor lattice mismatch between the (311)/(404) planes of MnFeO_4_ and the (101)/(111) planes of Ni(OH)_2_, MnFeO_4_ developed epitaxially along the Ni(OH)_2_ seeds during hydrothermal treatment. The creation of highly homogeneous nanosheet arrays with robust adhesion and linked morphology was made possible by this epitaxial growth method, as clearly shown in Figure 9A. With distinct lattice fringes verifying the epitaxial connection between MnFeO_4_ and Ni(OH)_2_, the HRTEM pictures demonstrated the single-crystalline structure of the MnFeO_4_ nanosheets, as shown in Figure 9B. Ion transport and electrochemical utilization were improved by the morphology’s wide surface area, open ion diffusion channels, and superior electrical contact. The MnFeO_4_ nanosheet arrays were therefore a promising electrode material for high-performance supercapacitors, delivering a high areal capacity of 302.6 mC cm^−2^ at one mA cm^−2^ and an energy density of 68.7 mWh cm^−2^ at 587 mW cm^−2^. The high-quality nanosheet structure is confirmed by Figure 9B, and the stepwise creation and phase transition during epitaxial growth are clearly explained by the schematic (Figure 9A). This study provides precise insights into how interface engineering can significantly enhance supercapacitor performance by highlighting the critical roles of phase transition and epitaxial growth control in shaping and adjusting the electrochemical behavior of metal nanoparticles.

### 3.5. Electron/Ion Beam Irradiation

Phase changes in nanomaterials can be triggered by localized energy from high-energy electron or ion beams, and these changes are often observed within TEM chambers. For example, in situ TEM studies have shown that localized heating and ligand removal cause Au nanosheets to transition from 2H to fcc structure when exposed to electron beam irradiation [34]. Other publications have demonstrated that beam-induced restructuring of Pd, Pt, and Au nanostructures under controlled irradiation confirms this as an effective method for phase control [55].

**Figure 10:**
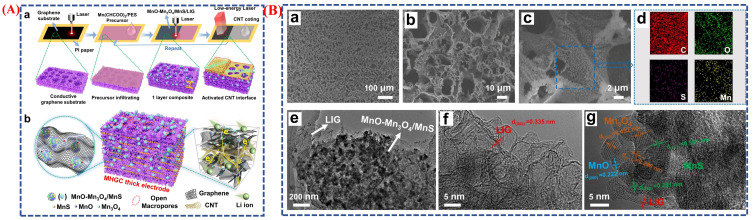
(**A**)—(**a**,**b**) Schematic process of high-conductivity MHGC electrode. (**B**) (**a**–**g**) Low- and high-magnification SEM images reveal the porous top-view structure of MnO–Mn_3_O_4_/MnS/LIG synthesized from a 0.4 mol L^−1^ Mn(CH_3_COO)_2_/PES precursor at an 8 W laser power. The high-resolution SEM confirms the uniform dispersion of MnO–Mn_3_O_4_/MnS heterostructures. Elemental mapping of the selected area verifies the homogeneous elemental distribution. TEM and HRTEM images further reveal the nanoporous LIG framework and well-defined MnO–Mn_3_O_4_/MnS heterostructure (reproduced with permission [56]).

Song et al. developed MnO–MnO_4_/MnS heterostructure-anchored porous graphene (LIG) thick electrodes with carbon nanotube (CNT) bridges using a distinctive laser-induced structural engineering technique [56], as shown in (Figure 10A(a,b)). During laser irradiation, manganese acetate and polyethersulfone (PES) polymer precursor simultaneously decompose and transform, as depicted in the schematic, which illustrates the phase transition process in a single-step laser induction. PES carbonizes into a three-dimensional porous graphene structure. At the same time, manganese acetate forms a MnO–MnO_4_/MnS multiphase heterostructure through initial conversion into MnO and MnO_4_ oxides, followed by partial sulfurization from sulfur-containing gases emitted by PES. This in situ phase change and heterostructure formation not only produce highly conductive graphene channels but also introduce surfaces and defects that enhance electrochemical activity and charge transfer. A 3D conductive scaffold arises from strong mechanical coupling and vertical electron pathways facilitated by CNT interlayers between each laser-induced layer. Morphological features in Figure 10B(a–g) confirm the effectiveness of this method: SEM and TEM images show a uniform porous structure with heterostructure nanoparticles evenly distributed within the graphene network. The nanoscale MnO–MnO_4_/MnS heterostructures provide numerous active sites for redox reactions, while well-connected macropores enable efficient electrolyte transport. The phase transition from precursor oxides to stable heterostructures is validated by high-resolution TEM images, which also reveal lattice fringes corresponding to different crystalline phases. This synergistic structure facilitates rapid ion diffusion and electron transport, resulting in excellent electrochemical performance, including a specific capacitance of 954.5 mF cm^−2^ and 92.3% cycling stability after 6000 cycles. Overall, the morphological data (Figure 10B) and schematic illustration (Figure 10A) clearly demonstrate how laser-induced synthesis can control phase changes in metal nanomaterials, achieving high conductivity, tunable morphology, and enhanced supercapacitor performance.

### 3.6. Mechanical Deformation

Mechanical deformation, such as stretching or compression, induces lattice strain and distortion, which can lead to phase transitions. The 4H-to-fcc transition in 4H-Au nanoribbons under external stress was observed using in situ TEM and molecular dynamics simulations, suggesting that strain energy might overcome activation barriers for atomic rearrangement [25]. This approach offers a mechanical method for studying the stability of metastable phases.

**Figure 11:**
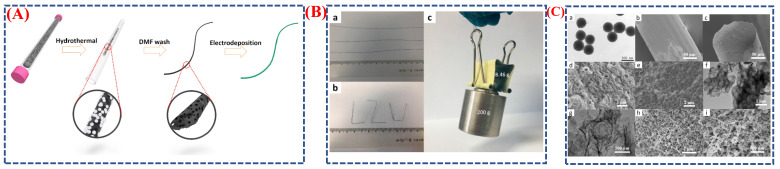
(**A**) Schematic illustration of the fabrication of the MGP electrode. (**B**) (**a**–**c**) digital image of MGP, with loading (**C**) (**a**–**i**) TEM, SEM images of MGP fiber (reproduced with permission [57]).

The work of Dong Liu et al. and others describes the creation of a PANI-coated microporous graphene fiber (PANI/MGF) through a simple in situ chemical polymerization process [57]. It demonstrates how structural design and phase changes influence flexibility and electrochemical behavior. The synthesis is schematically shown in Scheme (Figure 11A), beginning with the preparation of the microporous graphene fiber, followed by the application of a uniform PANI layer. During coating, aniline monomers undergo oxidative polymerization, forming PANI chains that strongly adhere to the graphene surface via π–π interactions. This phase transition from monomer to polymer establishes robust connections between the conductive graphene core and the PANI shell, enhancing charge transfer and structural stability. Morphological features in Figure 11B(a–c),C(a–i) confirm successful coating and structural modification. Unlike post-coating fibers, which have a continuous, uniform PANI layer with interconnected nanostructures, the SEM images reveal that the original graphene fibers have a rough, porous surface. The PANI layer maintains its porous structure, enabling efficient ion transport and mechanical flexibility, as seen in TEM and high-resolution images. By combining a pseudocapacitive PANI coating with a conductive graphene framework, outstanding electrochemical performance is achieved, including high specific capacitance, excellent rate capability, and durability under bending and stretching. This study highlights the importance of surface structural changes and phase transitions from monomer to polymer for achieving uniform morphology and improved charge storage. Therefore, the morphological images (Figure 11B,C) and schematic (Scheme) illustrate how interface engineering and controlled transformations in composite nanomaterials can lead to flexible, high-performance supercapacitors that combine exceptional electrochemical efficiency with mechanical resilience.

### 3.7. Other Approaches

In addition to thermal activation, aging, lithiation/delithiation, oxidation etching, electrochemical dealloying, high pressure, surface functionalization, secondary growth, irradiation, and mechanical deformation, these flexible phase-engineering techniques can be used in both mild and operational environments. For instance, amorphous/crystalline PdCu nanosheets exposed to ambient conditions over days gradually crystallize, with changes in binding energies and ligand desorption accompanying this process. This effectively adjusts the phase fraction without severe treatment [58]. Electrochemical cycling in intermetallic or alloy electrodes can reversibly induce phase transitions—such as between ordered and disordered states during battery operation, enabling dynamic phase adjustment [50]. Defective or amorphous areas may be removed through oxidation etching, which selectively oxidizes fewer stable regions, promoting reprecipitation onto more stable crystallites and enhancing crystallinity. Electrochemical dealloying further modifies the composition, phase distribution, and morphology by forming nanoporous gold from an Au–Ag alloy. This process involves dissolving the more active component from a parent alloy under controlled potential, resulting in a porous structure enriched in the noble phase [59]. This technique has been used to create nanoporous Cu_6_Sn_5_, a stable Li-ion anode for batteries or supercapacitors, which exhibits reversible phase transitions between Cu_6_Sn_5_ and Li_2_CuSn during cycling [60]. By allowing in situ or post-treatment control of phase, defect content, and active domain shape, these adaptable transformation methods complement more demanding physical techniques.

## 4. Phase Engineering in Transition Metal Dichalcogenide (TMD) Nanomaterials

The typical chemical formula for transition metal dichalcogenides (TMDs) is MX_2_, where M is a transition metal (groups IV–X) and X is a chalcogen element such as S, Se, or Te [61]. TMDs are a type of two-dimensional layered material with a structure similar to graphene. They exhibit a wide range of electrical, optical, mechanical, and magnetic properties because of their atomic organization and variable composition [62]. Depending on how the metal and chalcogen atoms are arranged and how the layers stack, TMDs can have different crystal structures, including hexagonal (2H), rhombohedral (3R), octahedral (1T), distorted octahedral (1T′), and orthorhombic (1Td) [63]. Interestingly, TMDs with the same chemical composition can have very different electrical characteristics depending on their phase. For example, the metasTable 1T and 1T′-MoS2 phases behave as metals and semimetals, respectively, while the sTable 2H-MoS2 phase is semiconducting [64]. However, due to their metastability and thermodynamic instability, producing high-quality, phase-pure 1T′-TMDs remains challenging; for instance, 1T′-MoS_2_ tends to revert to the 2H phase at around 60 °C [3].

Direct synthesis and phase transformation techniques are the primary methods researchers use to change the phase of TMD nanomaterials and overcome these limitations [65]. By using strategies like chemical vapor deposition (CVD), plasma-enhanced CVD, or solution-based approaches, direct synthesis aims to produce the metastable phase during growth. However, maintaining long-term stability and phase purity remains difficult [66]. In contrast, phase transformation methods utilize external stimuli such as mechanical strain, electron or laser irradiation, alkali-metal intercalation, or electrostatic gating to convert the sTable 2H phase into 1T or 1T′ phases [67]. Although transformation techniques are flexible, they often introduce faults and mixed phases. Over time, the metastable phase can revert to 2H [68]. To fully unlock TMDs’ potential in catalysis, electronics, and energy storage, controlled synthesis, defect reduction, and phase stabilization are crucial [69]. The direct synthesis strategy encompasses a wide range of methods, including colloidal/hydrothermal synthesis, gas–solid reaction, salt-assisted synthesis, and CVD.

Recent studies have demonstrated that precise control over the 1T–2H phase transition in TMDs can significantly impact their electronic and catalytic properties [70]. Additionally, comprehensive reviews have summarized strategies such as dopant-induced stabilization, strain engineering, and heterointerface modulation as effective ways to tune phase stability and charge transport in TMDs [71,72,73]. These findings underscore the importance of phase engineering not only for enhancing electrical conductivity but also for optimizing active site accessibility in TMD-based energy storage and conversion systems.

### 4.1. Colloidal/Hydrothermal Synthesis

The colloidal synthesis method has been widely used to produce TMD nanomaterials. Properly combining precursors, temperature, and gas atmosphere can effectively control the phase of the final products. Recently, more efforts have been focused on developing new solution-based synthesis methods to create TMD nanomaterials with unconventional phases.

Colloidal and hydrothermal/solvothermal synthesis are effective, scalable methods for creating electrode materials with precisely adjustable phase, defect population, morphology, and surface chemistry. These features are crucial for controlling pseudocapacitance, ion diffusion, and long-term cycling in supercapacitors and batteries. To enhance rate capability and structural reversibility in battery anodes/cathodes and pseudocapacitive electrodes, colloidal methods (wet-chemical, hot-injection, and ligand-assisted) provide atomistic control over nucleation and growth. This control allows for the synthesis of phase-selective polymorphs (e.g., 1T′ vs. 2H phases of TMD nanosheets), the engineering of surface ligands that stabilize metastable phases, and the regulation of size and shape to manage strain and surface-driven phase transformations during cycling [74,75]. To regulate the formation of crystallographic phases (such as spinel, layered, or tunneled oxides), establish hierarchical porosity, and evenly incorporate dopants or heteroatom features that increase accessible surface area, reveal active crystallographic facets, and adjust redox potentials for improved capacitance and capacity retention, hydrothermal and solvothermal routes utilize solvent chemistry, temperature, pressure, and mineralizers [76,77].

Both techniques are essential for phase engineering because they (1) enable the stabilization of metastable or low-symmetry phases at low temperatures through kinetic control, (2) allow for the controlled introduction of defects or oxygen vacancies to tailor electronic conductivity and redox kinetics via reducing/oxidizing solvothermal atmospheres or post-treatments, and (3) facilitate the formation of intimate heterostructures (core–shell, yolk–shell, mixed-phase nanocomposites) that combine stable bulk insertion behavior with fast surface redox (pseudocapacitance). These designs are practical for hybrid supercapacitors and high-rate battery electrodes [74,75,76,77]. Examples illustrating these capabilities include hydrothermally grown transition-metal oxides (MnO_2_, NiCo_2_O_4_, etc.), where phase (e.g., birnessite/todorokite/α-MnO_2_) and microstructure influence ion accessibility and cycling stability in aqueous pseudocapacitors [75,78], and colloidally synthesized TMD nanosheets in distinct 1T′ and 2H phases that exhibit markedly different lithiation behaviors as battery anodes [75]. When combined, hydrothermal and colloidal syntheses serve as both fabrication tools and phase-engineering platforms, enabling the deliberate selection, stabilization, and transformation of functional phases (including their defect chemistry) to enhance mechanical robustness, electrical conductivity, surface redox activity, and ion transport necessary for next-generation batteries and supercapacitors [74,75,76,77,78,79].

### 4.2. Gas–Solid Reaction

Although colloidal/hydrothermal synthesis methods can produce TMDs with unconventional phases in high yield, the products often exhibit limited phase purity and contain surface ligands that could be detrimental for many applications, especially for electronics. Gas–solid reaction is regarded as one of the most promising methods to prepare TMD crystals with unconventional phases.

**Figure 12:**
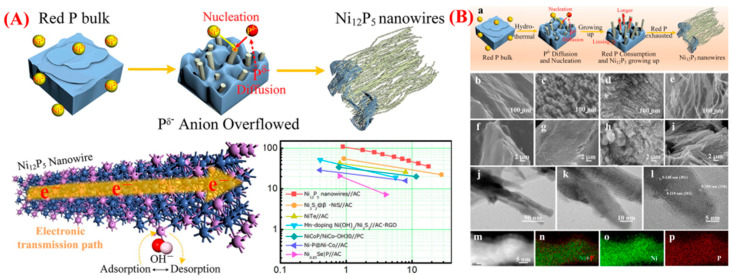
(**A**) Graphical abstract of Ni12P5 nanowires, (**B**) (**a**–**p**) Formation process diagram and morphological evolution of Ni_12_P_5_ nanowires at different reaction times (0, 1, 3, and 7 h). TEM and HRTEM images of the nanowires. STEM images with corresponding Ni and P elemental maps (Reproduced with permission [80]).

Yi Gan et al. demonstrated how controlled structural evolution and morphology design can enhance electrochemical performance by synthesizing Ni_12_P_5_ nanowires through a phosphorization-induced phase transition process [80]. The electrochemical concept and overall synthesis method are presented with a simple, visual description. The process involves preparing nickel-based precursors, followed by a thermal phosphorization treatment that converts them into crystalline Ni_12_P_5_ nanowires. During this stage, the precursor transforms from nickel hydroxide (Ni(OH)_2_) or nickel oxide (NiO) into the nickel phosphide phase (Ni_12_P_5_) in a solid-state phase transition. This transition significantly alters both the crystal structure and electrical properties, improving ion diffusion and conductivity. The resulting Ni_12_P_5_ nanowires form a densely connected, porous network that provides numerous electroactive sites and short pathways for charge transport, as further illustrated in the schematic of the graphical abstract. Figure 12A also shows the synthesis process and the morphological changes at different stages (Figure 12B(a–p)). After phosphorization, the precursor’s rough, nanorod-like surface gradually becomes smooth, crystalline Ni_12_P_5_ nanowires, indicating successful phase transition and structural refinement. Due to their large surface area and strong mechanical stability, these one-dimensional nanowires facilitate effective electron conduction and electrolyte penetration. When used as electrodes in battery–supercapacitor hybrid systems, these Ni_12_P_5_ nanowires exhibited excellent electrochemical performance, including high specific capacity, rapid charge–discharge capabilities, and improved cycle stability. The phase transformation from oxide/hydroxide precursors to metal phosphide nanostructures allows control of shape, enhancement of conductivity, and high-rate energy storage, as shown in the graphical abstract of Figure 12A.

### 4.3. Salt-Assisted Synthesis

Phase changes and morphology of electrode materials used in supercapacitors and batteries may now be effectively controlled by salt-assisted synthesis, which includes salt-template and molten-salt techniques. By offering a detachable salt framework, the salt-template technique directs nucleation and development, producing porous, high-surface-area structures. Molten salts function as ionic environments that stabilize complex phases or transformation paths, increase diffusion, and lower reaction temperatures. To stabilize MnO for supercapacitors and lithium-ion batteries and to provide hierarchical porosity, NaCl was used in the molten-salt-assisted development of MnO/biocarbon composites [81]. An analogous example of how the salt medium affects morphology and promotes structural changes is the production of oxygen-rich hierarchical porous carbon with exceptional supercapacitor performance. This is achieved by a molten-salt-assisted “self-activation” method employing NaCl/Na_2_CO_3_ [82]. Furthermore, NaCl nanoparticles were used in the salt-template synthesis of Fe–N–doped ultrathin carbon sheets to create ~5 nm thick layers with good capacitance, demonstrating how salt templates allow for ultrathin structures and improved electrochemical characteristics [83]. To convert the Na-birnessite precursor into rod-like NMO with high rate capability and stability, molten-salt-assisted synthesis of the layered oxide Na_0_._44_MnO_2_ for sodium-ion batteries employed NaCl/Na_2_CO_3_ [84]. Overall, the salt environment allows for fine control over morphology and encourages phase change under milder circumstances, resulting in conductive phases, high porosity, and quick ion/electron pathways, all of which are perfect for high-power and high-energy electrodes. Researchers may deliberately tailor the phase and morphology to maximize electrode performance for cutting-edge supercapacitors and batteries by adjusting the reaction parameters, salt type, and state (solid or molten).

### 4.4. CVD Synthesis

The bottom-up chemical vapor deposition (CVD) method is an effective way to grow one- and two-dimensional nanostructures and high-quality, conformal films directly on substrates. It is commonly used for making electrode materials for batteries and supercapacitors because it provides precise control over crystal quality, thickness, and shape [85,86,87,88,89,90]. In CVD, the solid phase develops gradually through the breakdown or reaction of gaseous precursors on a heated substrate, resulting in the formation of layers, wires, or forest-like structures. By tuning parameters such as temperature, pressure, precursor chemistry, and substrate type, researchers can selectively control phase (like different polymorphs of 2D transition metal dichalcogenides) and morphology (including monolayers, few-layer structures, vertical forests, nanotubes, and foams) [85,86,89]. Carbon nanotubes (CNTs) and CVD graphene form continuous, highly conductive networks with adjustable porosity and low defect levels, thereby enhancing the performance of carbon electrodes. These CVD-derived carbons enhance the rate capability and cycle stability of supercapacitors and hybrid devices by serving as superior current collectors and scaffolds for pseudocapacitive coatings, such as conducting polymers or metal oxides [87,88]. Vertical graphene, 3D graphene foams, and dense CNT arrays can be grown on metal foams or fibers using plasma-enhanced CVD (PECVD) or template-assisted CVD. This leads to mechanically durable, binder-free electrodes with large surface areas, which are vital for high power, stable energy storage, and the flexibility needed in wearable and deformable devices [88,90]. Moreover, by adjusting growth parameters such as temperature, chalcogen/metal partial pressure, and substrate strain, CVD allows for the synthesis or stabilization of non-equilibrium or mixed phases, such as controlled growth of metallic 1 T/1 T′ phases or mixed 1T/2 H domains in MoS_2_ and related TMDs. This can significantly boost conductivity and pseudocapacitive activity compared to the semiconducting phase [86,89]. CVD also enables direct deposition of metal oxides, phosphides, or sulfides onto 3D conductive substrates (like metal phosphide nanowires on Ni foam or oxide films), preserving nanomorphologies, forming low-resistance interfaces, and improving charge transfer and rate performance in batteries and hybrid supercapacitors [91,92]. A significant advantage of CVD is its ability to induce in situ phase changes such as controlled oxidation, phosphidation, or chalcogenization without damaging nanostructures, allowing active phase engineering to enhance electrochemical properties. Additionally, CVD techniques are scalable from lab to industry, suitable for roll-to-roll or template-assisted methods. They are versatile platforms for creating electrodes with tailored phases, crystal orientations, and hierarchical porosity, especially when combined with post-growth chemical modifications or templating, like 3D foams. These features lead to high specific capacitance, rapid charge/discharge cycles, and long cycle life in supercapacitors and batteries [87,88,90,93].

## 5. Phase Engineering in Metal-Organic Framework (MOF) Nanomaterials

As emerging porous materials, MOFs exhibit a diverse range of structures due to the various ways their metal nodes and organic linkers connect. Notably, even when metals and linkers are identical, the various coordination arrangements of metal atoms result in distinct topologies. Furthermore, within the same topology, the positioning of metal nodes and organic linkers can vary, owing to the flexibility of some linkers and the deformability of the framework.

**Figure 13:**
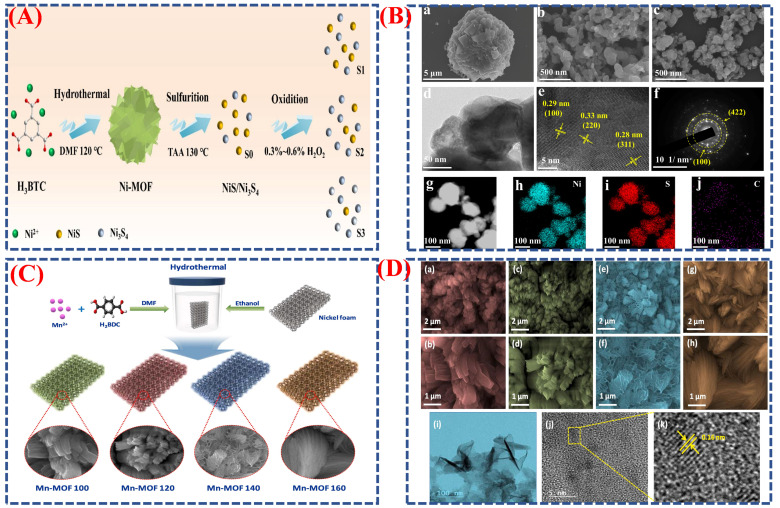
(**A**) Schematic of the preparation of Ni_3_S_4_ (**B**) (**a**–**j**) SEM images of Ni-MOF, SEM, TEM, and HRTEM, SAED of NiS/Ni_3_S_4_ samples, EDS mapping of NiS/Ni_3_S_4_, (**C**) Schematic preparation of Mn-MOF, (**D**) (**a**–**k**) FESEM, TEM, and HRTEM images of Mn-MOFs (reproduced with permission [94,95]).

Hongbo Tai et al. [94] investigate the enhancement of electrochemical performance in nanomaterials derived from metal–organic frameworks (MOFs) through phase engineering and morphological control. This investigation centers on the synthesis of NiS/Ni_3_S_4_ nanostructures derived from Ni-MOF precursors, aiming to improve crystal phases and elevate the performance of battery-type supercapacitors. Figure 13A illustrates the sequential synthesis process, beginning with the formation of microsphere-shaped Ni-MOFs, followed by a solvothermal sulfuration reaction that produces NiS/Ni_3_S_4_ nanoparticles (S_0_), and concluding with an oxidation treatment using H_2_O_2_ to modify the phase ratios, leading to optimized samples (S_2_). This phase-controlled approach demonstrates how gentle oxidation can alter the balance between NiS and Ni_3_S_4_ phases, resulting in the formation of heterojunction surfaces that enhance charge transfer and redox activity. Figure 13B(a–j) presents the morphological evolution of the materials as observed through SEM, TEM, and HRTEM analyses. The Ni-MOF precursor (Figure 13B) exhibits uniform microspheres (~8 μm), which transform into nanoparticles (~100 nm) with a “stone-like” morphology upon sulfuration Figure 13B(a–j). The optimized S_2_ sample maintains this nanostructure, showcasing rougher surfaces and clear crystalline interfaces aligned with NiS and Ni_3_S_4_ lattice planes. The elemental mapping Figure 13B(a–j) confirms the uniform distribution of Ni, S, and C, indicating successful conversion and compositional uniformity. The morphological enhancement achieved through MOF templating and oxidation results in an increased surface area (79.6 m^2^ g^−1^), which promotes rapid ion diffusion and provides numerous active sites, thereby directly improving the charge–discharge characteristics of the battery type. The enhanced S_2_ electrode demonstrates a specific capacitance of 2572 F g^−1^ at 2 A g^−1^, exceeding earlier samples due to its synergistic phase interfaces and improved electron transport pathways.

Pragati et al. investigated how phase transitions and shape changes influence enhanced energy storage in manganese-based MOF nanostructures [95]. They created Mn-MOF using an easy hydrothermal process, as shown in Figure 13C,D(a–k), where the temperature and precursor concentration primarily determined the crystalline phase and shape. During synthesis, a two-dimensional sheet-like structure appeared as the material shifted from an amorphous coordination network to a crystalline layered MOF. This hierarchical nanosheet design, with its large surface area, numerous redox-active sites, and short ion diffusion paths, was crucial for achieving high capacitance and fast charge–discharge performance. Controlling phase transitions within the MOF lattice helps preserve structural integrity during cycling, ensuring long-term durability, as illustrated by schematic diagrams and microscopic images. In conjunction with the research by Li et al., these results demonstrate that the phase engineering of metal MOF nanoparticles effectively tunes the electrical properties, shape, and crystal phase of advanced electrode materials for supercapacitors and batteries.

## 6. Electrochemical Energy Storage in Phase Engineering Nanomaterials

Electrochemical energy storage technologies, like rechargeable batteries, lithium-sulfur (Li–S) batteries, and supercapacitors, are gaining prominence due to their crucial role in enabling portable electronics, electric vehicles, and renewable energy systems. Employing phase engineering in nanomaterials has emerged as a revolutionary strategy to boost their performance and durability. This technique involves deliberately altering the crystal structure and phase composition of nanomaterials to improve properties such as conductivity, stability, and capacity. Such modifications have proven successful in addressing the limitations often encountered by traditional materials in energy storage applications. To ensure the reliability of the experimental data, uncertainty analysis was performed for all key measurements. The possible sources of error included instrumental precision, measurement repeatability, and environmental fluctuations. Each electrochemical test was conducted at least three times under identical conditions, and the average values, along with their corresponding standard deviations (±σ), are reported. The overall experimental uncertainty was estimated to be within ±3–5% for electrochemical measurements (specific capacitance, capacity, and energy density) and ±2% for structural characterization results (XRD peak position, BET surface area, etc.). These values confirm the consistency and reproducibility of the obtained results.

### 6.1. Rechargeable Batteries

Rechargeable batteries, particularly lithium-ion (Li-ion) types, are crucial for modern energy storage solutions. They operate through the reversible transfer of lithium ions between the anode and cathode, allowing for energy storage and discharge. However, challenges such as low energy density, thermal instability, and degradation with use persist. Enhancing the structural robustness and electrochemical properties of electrode materials is crucial for improving battery performance and durability. The electrochemical performance of the synthesized phase-engineered materials was evaluated using a standard CR2032-type coin cell configuration. The anode consisted of the active material (phase-engineered nanostructure) mixed with conductive carbon black and polyvinylidene fluoride (PVDF) binder in an N-methyl-2-pyrrolidone (NMP) solvent at a weight ratio of 8:1:1, coated on copper foil. The cathode was lithium metal foil. A microporous polypropylene (Celgard 2400) membrane was used as the separator, and 1 M LiPF_6_ dissolved in a 1:1 (*v*/*v*) mixture of ethylene carbonate (EC) and dimethyl carbonate (DMC) served as the electrolyte. The nominal voltage of the assembled coin cell was approximately 3.7 V, with a nominal capacity of around 900–950 mAh g^−1^, depending on the active material composition and structure.

### 6.2. Metal-Ion Batteries

Metal-ion batteries, such as Li-ion, sodium-ion, and potassium-ion batteries, have been widely studied for their potential in large-scale energy storage. The performance of these batteries heavily depends on the structural features of the electrode materials. Phase engineering of nanomaterials has been employed to modify the crystal structures of electrode materials, thereby enhancing ionic conductivity and improving structural stability during charge and discharge cycles.

**Figure 14:**
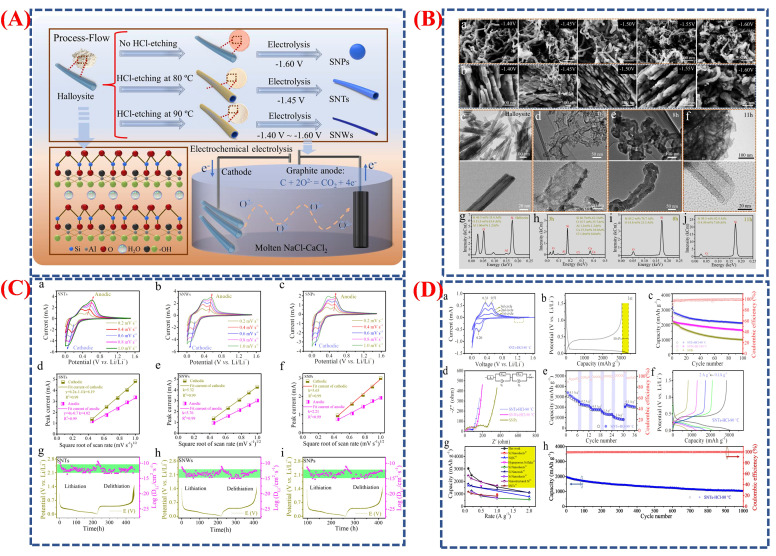
(**A**) Schematic illustration of different Si nanomaterials, (**B**) (**a**) SEM of original halloysite; (**b**,**c**) SEM after HCl etching at 80 °C (24 h) and 90 °C (12 h); (**d**–**f**) SEM of electrolysis products at −1.45, −1.55, and −1.60 V (11 h); (**g**–**j**) TEM of SNTs, SNWs, and SNPs formed under corresponding conditions. (**C**) (**a**–**f**) CV curves and peak current–square root relationships of SNTs, SNWs, and SNPs electrodes; (**g**–**i**) GITT curves and Li⁺ diffusion coefficients of SNTs, SNWs, and SNPs electrodes. (**D**) (**a**) CV curves; (**b**) Initial charge–discharge profiles; (**c**) Cycling performance; (**d**) EIS plots; (**e**) Rate performance; (**f**) Discharge–charge curves at various current densities; (**g**) Rate performance comparison with reported Si-based materials; and (**h**) Long-term cycling stability of the SNTs electrode. (reproduced with permission [94]).

Wang et al. demonstrate the synthesis of various silicon (Si) nanostructures, including nanotubes (SNTs), nanowires (SNWs), and nanoparticles (SNPs), through a controlled electrochemical process [96]. They explore how the shape of these structures affects the performance of lithium-ion batteries (LIBs). Silicon’s high theoretical capacity (up to 3580 mAh g^−1^) makes it a promising anode material. Still, a significant challenge is its ~400% volume expansion during lithiation and delithiation, leading to cracking and capacity loss. Morphological engineering provides solutions, offering insights into how one-dimensional and hollow Si structures can enhance cycle stability and capacity. The process of fabricating different Si nanostructures from halloysite clay (Al_2_(OH)_4_Si_2_O_5_·nH_2_O) via molten salt electrolysis is shown in Figure 14A. The electrolysis voltage and HCl etching temperature control the final shape of nanotubes, nanowires, or nanoparticles. For example, nanotubes form at 80 °C with a potential of −1.45 V, nanowires at 90 °C, and nanoparticles when the clay is unetched at −1.60 V. This synthesis approach highlights the importance of tuning reaction parameters. Figure 14B(a–j) outlines the key steps in forming these nanostructures, with SEM and TEM images confirming their morphology under various electrolysis conditions. During electrolysis, halloysite transitions into Si nanotubes over time, showing an intermediate sheet-like stage that bends into tubes as surface energy decreases. Unlike nanowires, which form via dissolution–deposition, Si nanotubes develop through a distinct sheet-to-tube transformation.

The electrochemical performance of SNPs, SNWs, and SNTs as LIB anodes is shown in Figure 14C(a–i). Charge–discharge curves confirm their high capacity and reversibility, while cyclic voltammetry (CV) profiles illustrate the lithiation and delithiation processes. The SNT electrode, with a Coulombic efficiency of 89.6%, starts with an initial charge capacity of 3044 mAh g^−1^. SNTs outperform SNWs and SNPs, as seen in the cycling data (Figure 14C) which retains 2021 mAh g^−1^ after 100 cycles, compared to 1550 mAh g^−1^ and 911 mAh g^−1^ for SNWs and SNPs, respectively. Furthermore, EIS and rate capability results (Figure 14C(e,f)) show enhanced ion transport and lower charge-transfer resistance for SNTs. This exceptional performance is attributed to the hollow design, providing a large surface area (99.9 m^2^ g^−1^), short Li^+^ diffusion paths, and space for volume expansion. The kinetic and diffusion properties of the three Si electrodes are analyzed in Figure 14D(a–h). CV, GITT, and Li^+^ diffusion coefficient calculations reveal that SNTs have significantly higher Li^+^ diffusivity than SNWs and SNPs (2.31 × 10^−9^ to 1.51 × 10^−13^ cm^2^ s^−1^). Faster charge transfer and stable cycling are supported by the thin tubular walls and open channels, which improve kinetics and reduce diffusion distances (around 6 nm for SNTs). The impressive long-term cycling stability of SNTs, maintaining 1033 mAh g^−1^ even after 1000 cycles at 1 A g^−1^, is further explained by these kinetic advantages. Overall, the research provides a detailed understanding of how electrochemical activity correlates with morphogenesis. Compared to nanowires and nanoparticles, silicon nanotubes offer higher lithium storage capacity, better diffusion kinetics, and enhanced mechanical stability due to their hollow, sturdy structure. As shown in this study, optimizing morphology through phase and electrochemical engineering is crucial for developing high-performance silicon anodes for next-generation lithium-ion batteries.

**Figure 15:**
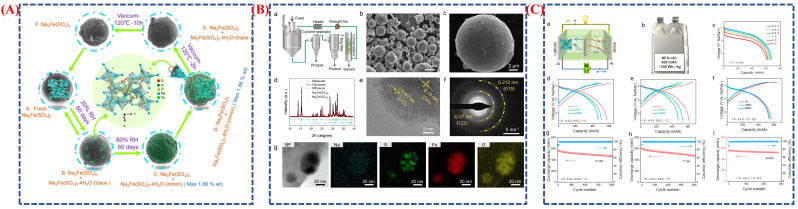
(**A**) Dynamic air-stability mechanism of the NFS cathode material. (**B**) (**a**) Schematic of NFS preparation via spray-drying; (**b**,**c**) SEM images; (**d**) XRD patterns with Rietveld refinement; (**e**) HRTEM image; (**f**) SAED pattern; and (**g**) TEM-EDS elemental mapping of NFS. (**C**) (**a**) Schematic of the Na-ion full cell; (**b**) Photograph of the NFS–HC pouch cell (22.9 g); (**c**) Rate performance (1.5–4.2 V); (**d**–**f**) Voltage profiles at various voltage ranges and C-rates (The C-rate, which represents the charge and discharge current relative to the electrode’s theoretical (or nominal) capacity, indicates how fast a battery is charged or discharged (e.g., 1C corresponds to a full charge or discharge in one hour)); and (**g**–**i**) Corresponding discharge–cycle plots (reproduced with permission [97]).

The widespread availability, low cost, and abundance of sodium resources have made sodium-ion (Na-ion) batteries an increasingly attractive alternative to lithium-ion batteries in recent years. These batteries are particularly suitable for low-speed electric vehicles and large-scale energy storage, where safety and affordability outweigh energy density. In this study, researchers developed a Na_2_Fe(SO_4_)_2_ (NFS) cathode, demonstrating its long cycle life, structural reversibility, and excellent air stability [97]. The NFS cathode was created through spray-drying, producing uniform spherical particles with smooth surfaces and an average diameter of about 4 µm, as shown in Figure 15A. SEM, XRD, and TEM analyses reveal that the synthesized NFS possesses a well-crystallized structure with evenly distributed Na, Fe, and O, alongside minor Na_6_Fe(SO_4_)_4_ impurities. This morphology promotes ion transport and helps maintain structural stability over multiple charge–discharge cycles. Figure 15a,b show the long-term electrochemical performance and the practical battery setup, providing insights into electrochemical behavior and air stability in real-world conditions. The schematic in Figure 15a illustrates the self-limited, reversible surface hydration process that NFS particles undergo in ambient humidity. At low humidity (20% RH), surface hydration is minimal, preserving a stable core–shell structure. At higher humidity (60% RH), a thin hydrated layer (Na_2_Fe(SO_4_)_2_·4H_2_O) forms, protecting the bulk material from further degradation. The material’s “dynamic air stability” is confirmed by the reversible hydration process during electrode drying. The performance of the NFS hard carbon (HC) pouch cell is shown Figure 15B(a–g), retaining 81.9% of its capacity after 1000 cycles, with an average voltage of 3.58 V and a specific energy near 100 Wh/kg. These results underline the enhanced structural and electrochemical stability of the NFS cathode. The morphology in Figure 15B supports efficient sodium-ion diffusion, while the hydration mechanism in Figure 15B improves air stability. The high energy density and extended cycling performance depicted in Figure 15C(a–i) emphasize the potential of NFS-based sodium batteries as cost-effective, environmentally sustainable energy storage solutions.

### 6.3. Lithium-Sulfur Batteries

Li–S batteries’ high theoretical energy density and abundant sulfur supply make them very promising for future energy storage. However, issues like the polysulfide shuttle effect and poor sulfur conductivity pose challenges for practical use. By altering the sulfur host structures, phase engineering of nanomaterials offers strategies to improve conductivity and reduce polysulfide dissolution.

**Figure 16:**
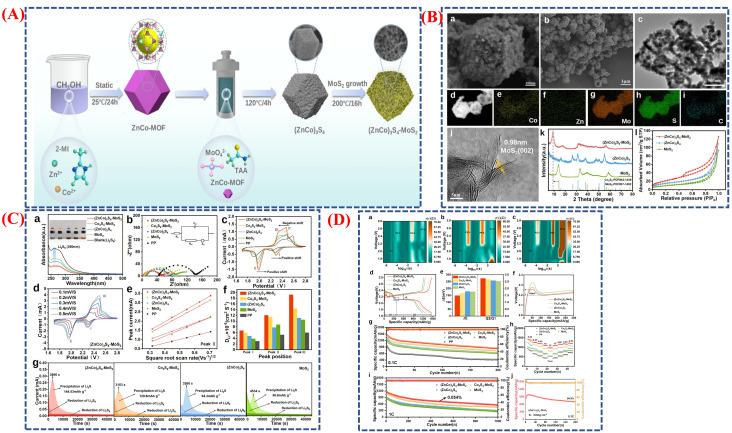
(**A**) Flow chart of (ZnCo)_3_S_4_–MoS_2_, (**B**) (**a**,**b**) SEM images; (**c**,**j**) HRTEM images; (**d**–**i**) SEM-EDS elemental mapping; (**k**) XRD patterns; and (**l**) N_2_ adsorption–desorption isotherms of (ZnCo)_3_S_4_–MoS_2_, (ZnCo)_3_S_4_, and MoS_2_. (**C**) (**a**) UV–vis spectra and Li_2_S_6_ adsorption; (**b**) Nyquist plots with equivalent circuit; (**c**,**d**) CV curves; (**e**) Peak current vs. log(scan rate); (**f**) Li⁺ diffusion coefficients; and (**g**) Li_2_S nucleation profiles for (ZnCo)_3_S_4_–MoS_2_, Co_3_S_4_–MoS_2_, (ZnCo)_3_S_4_, and MoS_2_. (**D**) (**a**–**c**) In situ DRT contour maps; (**d**) Charge–discharge profiles at 0.1 C; (**e**) ΔE and Q_2_/Q_1_ values; (**f**) Enlarged charging curve; (**g**) Cycling performance at 0.1 C; (**h**) Rate performance; (**i**) Cycling stability at 1 C; and (**j**) High-sulfur-loading cycling performance of (ZnCo)_3_S_4_–MoS_2_-based Li–S batteries (Reproduced with permission [98]).

Lithium-sulfur (Li–S) batteries are among the most promising next-generation energy storage options due to their low cost, high specific capacity (1675 mAh g^−1^), and high theoretical energy density (2600 Wh kg^−1^). Nevertheless, challenges such as slow redox reactions of polysulfides, shuttle effects, and volume expansion during cycling hinder their practical application. Tingting Zhao and team created a (ZnCo)_3_S_4_–MoS_2_ heterostructure to tackle these issues by modifying the separator and controlling morphology, significantly improving Li–S battery performance [98]. Their two-step synthesis involves in situ growth of MoS_2_ nanosheets on 3D (ZnCo)_3_S_4_ structures derived from ZnCo-MOF precursors. Figure 16A shows the entire fabrication process of this heterostructure. The design fosters close contact between the sulfides, generating an electric field at the heterointerface that boosts charge transfer and polysulfide conversion. Figure 16B(a–i) provides morphological and structural analyses, revealing uniform MoS_2_ nanosheets covering the (ZnCo)_3_S_4_ surface and the preservation of its hollow dodecahedral shape, as seen in SEM and HRTEM images. Its mesoporous structure and large surface area (78.5 m^2^ g^−1^) enhance catalytic kinetics and cycle stability by enabling efficient ion and electron transport and offering numerous active sites for polysulfide adsorption.

Figure 16C(a–g) illustrates the effects of separator modification and electrochemical testing, emphasizing the excellent Li^+^ diffusion and polysulfide trapping capabilities of the (ZnCo)_3_S_4_–MoS_2_-modified separator. UV-vis spectra verify superior polysulfide adsorption, while Nyquist plots and CV curves indicate improved redox kinetics and a lower charge transfer resistance of 51.46 Ω compared to standard samples. The heterostructure’s inherent electric field promotes bidirectional polysulfide catalysis, ensuring efficient conversion of soluble intermediates into insoluble Li_2_S during discharge. The overall electrochemical performance is summarized in Figure 16D(a–i), where the Li–S cell with the (ZnCo)_3_S_4_–MoS_2_/PP separator achieves a high initial capacity of 1459.9 mAh g^−1^ at 0.1 C and maintains 837.9 mAh g^−1^ after 200 cycles, with an 84.9% capacity retention. It also demonstrates excellent long-term stability and high-rate performance, maintaining 561.7 mAh g^−1^ after 1000 cycles at 1 C. These enhancements result from combined Zn doping, a 3D hollow structure, and heterointerface-induced electric fields, which improve polysulfide immobilization, minimize shuttle effects, and speed up charge transfer. Overall, this study highlights the critical role of morphological design and heterostructure engineering in advancing Li–S batteries toward practical, high-performance use. Table 2 compares various battery materials, like Li-ion, Na-ion, and Li-S, their specific capacities, and energy densities.

### 6.4. Supercapacitors

Supercapacitors, also known as ultracapacitors, store energy through the electrostatic charge buildup on electrode surfaces, delivering high power density and enabling fast charge–discharge cycles. However, their energy density is lower than that of batteries. Phase engineering of nanomaterials can improve the electrochemical performance of supercapacitors by optimizing the structure and composition of electrode materials to boost surface area and conductivity.

**Figure 17:**
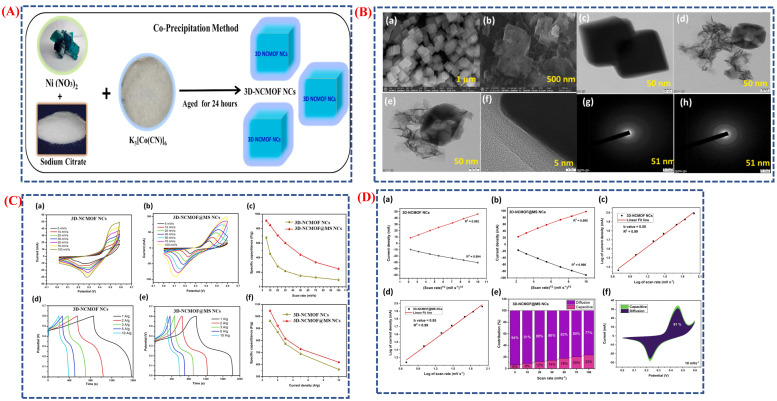
(**A**) Schematic illustration of 3D-NiCo-MOF, (**B**) (**a**,**b**) HR-SEM images; (**c**–**f**) HR-TEM images; and (**g**,**h**) SAED patterns of 3D-NCMOF and 3D-NCMOF@MS nanocomposites. (**C**) (**a**,**b**) CV curves; (**c**,**d**) GCD profiles of 3D-NCMOF and 3D-NCMOF@MS NCs; (**e**,**f**) comparative specific capacitance from CV and GCD analyses. (**D**) (**a**,**b**) Linear fitting; (**c**,**d**) b-values of redox peaks for 3D-NCMOF and 3D-NCMOF@MS NCs; (**e**) Diffusion contribution at different scan rates; and (**f**) Battery behavior contribution at 10 mV s^−1^ for 3D-NCMOF@MS NCs (reproduced with permission [113]).

Supercapacitors are advanced energy storage devices that combine high power density, fast charging and discharging capabilities, and a long cycle life. They bridge the gap between traditional capacitors and rechargeable batteries. These devices are essential for delivering quick energy bursts and maintaining stability over thousands of cycles, making them ideal for renewable energy sources, electric vehicles, and portable gadgets. In this study [113], 3D-NCMOF@MS nanocubes were developed as effective electrode materials for battery-type supercapacitors. This was achieved by integrating a three-dimensional nickel–cobalt metal–organic framework (3D-NCMOF) with two-dimensional molybdenum disulfide nanosheets (2D-MS). Scheme (Figure 17A) illustrates the formation of these nanocubes, where the 3D-NiCo-MOF serves as a template, and MoS_2_ nanosheets grow uniformly on its cubic surface through a solvothermal process. This approach enhances ion dispersion and charge storage by providing a large surface area and improved electrical conductivity. HR-SEM and HR-TEM images of the interconnected porous nanocubes, detailed in Figure 17B(a–h), confirm the uniform cubic structure of 3D-NCMOF and successful coating with MoS_2_ nanosheets. These structural features facilitate ion transport and electrolyte penetration, leading to improved electrochemical performance. Due to the synergistic interaction between the MOF framework and MoS_2_ layers, 3D-NCMOF @ MS outperforms the pristine 3D-NCMOF, as shown in Figure 17C(a–f), with clear redox peaks and a high specific capacitance of 1048 F g^−1^ at 1 A g^−1^. Longer discharge times and improved redox reversibility indicate a strong pseudo-capacitive effect. Figure 17D(a–f) explores the charge storage mechanism, confirming a diffusion-controlled redox process through linear Randles–Sevcik plots and b-values. At 10 mV s^−1^, approximately 94% of ion diffusion accounts for the total, indicating battery-like behavior. Electrochemical impedance spectra and cycling data (Figure 17C) demonstrate excellent long-term stability, retaining 99. 06% of capacitance after 5000 cycles, with a very low charge transfer resistance of 0.39 Ω cm^−2^. Compared to similar materials, its energy density exceeds 188.64. 64 Wh kg^−1^, and its power density surpasses 400 W kg^−1^, demonstrating superior performance. Overall, the 3D-NCMOF @ MS composite shows promise for next-generation supercapacitors. It features an optimized electrode design with engineered morphology and strong synergistic effects that significantly improve electrochemical conductivity, stability, and energy storage capacity.

## 7. Conclusions

This review highlights the essential integration of phase engineering and morphological control to enhance the electrochemical properties of nanomaterials. The methods of direct synthesis and phase transformation enable the creation of unconventional or mixed phases, which exhibit distinctive electronic structures and enhanced electrochemical performance. The relationship between morphology and phase structure plays a crucial role in influencing surface area, ion diffusion, and electron transport, all of which are essential factors in evaluating the performance of batteries and supercapacitors. The management of phase modification in both metallic and TMD-based nanomaterials has demonstrated enhancements in energy storage capacity, rate performance, and cycling stability. The optimized phase-engineered ZnCo_2_O_4_@Ti_3_C_2_ composite electrode delivered a high specific capacitance of 1013.5 F g^−1^, excellent cyclic stability with over 85% retention after 10,000 cycles, and a superior energy density of 42.8 Wh kg^−1^ at a power density of 750 W kg^−1^. These findings confirm that controlled phase engineering and structural modulation can effectively enhance charge transport and electrochemical stability, offering promising potential for next-generation energy storage applications. The findings presented in this summary indicate that customizing crystal phases alongside morphology represents a viable approach for enhancing energy storage characteristics. Ongoing initiatives aimed at enhancing phase-morphology control and material design are poised to propel the evolution of high-performance batteries and supercapacitors, which are essential for the advancement of future energy technologies.

Funding: This study was supported by the National Research Foundation of Korea (grant number: NRF-2021R1A2C1008272). This work was supported by the Ministry of Trade, Industry and Energy (KEIT) under the project title "International standard development of evoluation methods for nano-carbon-based high-performance supercapacitors for electric vehicles" (project#20016144).

## Figures and Tables

**Table 1:** Summary of a few representative works on the direct synthesis of unconventional phase nanomaterials.

Synthesis Method	Metal	Unconventional Phase	Reference
In situ growth on graphene-oxide template (solution)	Au	Hexagonal close-packed (hcp, 2H) Au square sheets	[24]
High-yield colloidal (solution) synthesis of nanoribbons	Au	4H hexagonal Au (4H Au nanoribbons)	[25]
Solution-phase epitaxial coating on 4H Au template	Ir, Rh, Os, Ru, Cu	4H hexagonal (epitaxial 4H Ir, Rh, Os, Ru, Cu)	[26]
Ligand-protected single-crystal cluster synthesis (X-ray)	Au (nanocluster)	Body-centered cubic (bcc) Au_38_ nanocluster	[27]
Atomically precise clusters/spectroscopy	Au (atom-precise NCs)	hcp Au_30_ and bcc Au_38_ nanoclusters	[28]
Solvothermal/electron-beam decomposition	Rh	Hexagonal close-packed (hcp) Rh nanoparticles	[29]
Liquid-cell in situ TEM (H supply control)	Pd → Pd hydride	Metastable hcp palladium hydride (PdH_x_)	[30]
Chemical reduction/shape control (citric acid, seed-mediated)	Ag	Metastable hexagonal polytypes of Ag (2H/4H)	[31]
Template-free colloidal growth (nanoplates)	Ag	2H and related metastable Ag structures	[32]
DC magnetron/high-pressure sputtering	Ag	Unusual hexagonal (4H) Ag observed	[33]
Gas + e-beam in situ TEM	Au	fcc → metasTable 4H phase	[34]
Pechini/sol–gel heat treatment	Ni	Hexagonal close-packed (hcp) Ni nanoparticles	[35]
Colloidal/polyol/PEG reduction	Ni	hcp Ni synthesized in colloidal/PEG systems	[36]

**Table 2:** Comparison of Li-ion, Li–S, and Na-ion electrode materials, theoretical and practical specific capacities, typical energy densities, stability characteristics.

No.	Material	Specific Capacity (mAh g^−1^)	Energy Density (Wh kg^−1^)	Stability/Notes	Reference
1	LiCoO_2_	150	555.0	Typical practical values: retained capacity ~150 mAh g^−1^.	[99]
2	LiFePO_4_ (carbon/graphene-modified)	208	707.2	High reversible capacity; graphene-modified electrode.	[100]
3	NMC811 (LiNi_0_._8_Mn_0_._1_Co_0_._1_O_2_)	≈200	760.0	Depends on rate and SOC window; first-cycle losses are common.	[101]
4	NCA (LiNi-Co-Al)	≈200	740.0	High capacity; stability improved by Al doping.	[102]
5	LNMO/LiNi_0_._5_Mn_1_._5_O_4_	147	690.9	High voltage (~4.7 V); electrolyte decomposition limits cycle life.	[103]
6	Graphite	372	37.2	Excellent cyclability; baseline anode.	[104]
7	Si–C encapsulated Si (SF@G)	2646/2194	1058.4/877.6	Stable 500 cycles; high Coulombic efficiency.	[105]
8	Li metal (theoretical)	3860	—	Very high capacity; dendrite and CE limitations.	[106]
9	Li–S (sulfur cathode)	1661	3488.1	High capacity; shuttle effect mitigated by host design.	[107]
10	Na_3_V_2_(PO_4_)_3_ (NVP)	110	374.0	Stable cycling; good ion conductivity.	[108]
11	Na_0_._44_MnO_2_	108	324.0	Good rate capability; moderate cycle retention.	[109]
12	Hard carbon (Na anode)	250–350	—	Common Na-ion anode; 300+ cycles with 80–90% retention.	[110]
13	LiMn_2_O_4_ (spinel)	147	588.0	Good power; capacity fades at high T/current.	[111]
14	NMC811 (operando studies)	159–200	606.4–760.0	Studied diffusion limits and phase evolution.	[112]
15	Na_0_._44_MnO_2_/other Na cathodes	100–120	300–420	Excellent cycling (e.g., 105 → 102 mAh g^−1^ after 100 cycles).	[109]

## Nomenclature, Greek Symbols, Subscripts, Superscripts, Acronyms, and Abbreviations

**Symbol**	**Definition/Description**
C	Specific capacitance (F g^−1^ or mF cm^−2^)
E	Energy density (Wh kg^−1^)
P	Power density (W kg^−1^)
V	Voltage (V)
I	Current (A or mA)
Q	Charge or capacity (C or mAh g^−1^)
t	Time (s or h)
R	Resistance (Ω)
σ	Electrical conductivity (S cm^−1^)
η	Efficiency (%)
d	Thickness or diameter (nm or µm)
